# Editorial: Biomarkers, functional mechanisms, and therapeutic potentials in gastrointestinal cancers

**DOI:** 10.3389/fonc.2023.1276414

**Published:** 2023-10-26

**Authors:** Jun Huang, Qun Zhang, GuangZhao Pan, Xin Hu, Dongshi Chen, Kui Zhang

**Affiliations:** ^1^ School of Life Sciences, Zhengzhou University, Zhengzhou, Henan, China; ^2^ Department of Respiratory and Critical Care Medicine, First Affiliated Hospital, Nanjing Medical University, Nanjing, China; ^3^ Zhejiang Cancer Hospital, Hangzhou Institute of Medicine (HIM), Chinese Academy of Sciences, Hangzhou, Zhejiang, China; ^4^ Key Laboratory of Prevention, Diagnosis and Therapy of Upper Gastrointestinal Cancer of Zhejiang Province, Hangzhou, Zhejiang, China; ^5^ State Key Laboratory of Resource Insects, Medical Research Institute, Southwest University, Chongqing, China; ^6^ Department of Medicine, Keck School of Medicine of University of Southern California (USC), Los Angeles, CA, United States; ^7^ The Pritzker School of Molecular Engineering, The University of Chicago, Chicago, IL, United States

**Keywords:** gastrointestinal cancers, biomarker, therapeutic strategy, drug resistance and metastasis, clinical studies

## 1 Introduction

Gastrointestinal cancers are becoming increasingly serious health concerns, with rising incidence rates due to shifts in dietary habits, environmental pollution, and other risk factors (1, 2). The current primary therapeutic approaches for gastrointestinal cancer include surgery, chemotherapy, radiotherapy, and more recently, immunotherapy (3, 4). Although remarkable progresses have been achieved, the treatment of those malignancy diseases still faces significant challenges, including tumor recurrence, metastasis, and drug resistance (5, 6). The advent of cutting-edge research technologies, such as genomics, high-throughput sequencing, proteomics, metabolomics, immunotherapy, nanotechnology, liquid biopsy, robotic surgery, artificial intelligence, organoid modeling, and microbiome analysis, has sparked a rapid transformation in clinical and biomedical research. These cutting-edge approaches have led to discovery of numerous new biomarkers, therapeutic targets, diagnostic tools, treatment strategies, underlying biological pathways, and potential mechanisms for understanding and combating gastrointestinal cancers (7–13).

The latest research findings, utilizing both classical methodologies and innovative approaches, have the potential to deepen and refine our understanding of gastrointestinal cancers. These discoveries shed new light on existing theories, offering fresh perspectives, and even subtly modifying long-established views and conclusions. They may open new avenues of understanding, leading to more targeted and effective treatments with acceptable advance events for patients. The timely collection and prospective reviews on the latest discoveries of new diagnostic/prognostic factors, biomarkers, and risk factors will facilitate the identification of novel clinical indicators and enhance our understanding of the key molecular mechanisms underlying cancer initiation, progression, recurrence, and drug resistance. Furthermore, the exploration of the targeted anti-cancer agents will provide new and effective supports to traditional chemotherapy and radiotherapy in the treatment of gastrointestinal cancers. Overall, these efforts to explore the Research Topic of Biomarkers, Functional Mechanisms, and Therapeutic Potentials in Gastrointestinal Cancers will contribute to improving the prognosis and survival of cancer patients.

## 2 Identification and exploration of key proteins, genes, and molecular markers

The study on protein level has emerged as a pivotal approach, offering new insights into disease mechanisms, paving the way for innovative diagnostics, and highlighting targeted therapeutic opportunities (14). Gu et al. identified proteins from extracellular vesicles to create a logistic regression classifier that distinguishes gastric adenocarcinoma from healthy samples, particularly identifying advanced stages, highlighting the diagnostic potential of exosome-derived proteins. Xu et al. developed a mass spectrometry method to accurately quantify HER2 protein in advanced gastric adenocarcinoma, overcoming traditional method limitations. With clinical validation, this technology could be a significant tool for gastric cancer detection. Those studies utilize mass spectrometry to identify and quantify specific proteins, enhancing diagnostic accuracy in cancer detection.

The ongoing search for accurate diagnosis and prognosis in gastric cancer has led to the investigation of novel biomarkers. Understanding these biomarkers improves our understanding of the complex nature of the disease and opens the door to more targeted and effective treatment strategies. Zhang et al. created a method for detecting plasma tRF-33, a novel biomarker for gastric cancer. It showed a gradient change across gastric lesions and correlated with various factors, such as differentiation stage, tumor size. It could distinguish between early and advanced stages and was associated with unfavorable outcomes, highlighting its potential as a critical tool for monitoring and managing gastric cancer progression. Wu et al. studied highly mobile group (HMG) proteins in gastric cancer, finding that specific HMGs correlated with survival rates and disease-free survival, and could distinguish cancer from normal tissues. Their expression was also linked to immune cell infiltration levels, highlighting their potential as diagnostic markers, and hinting at a complex relationship with the disease. Ding et al. discovered that increased GLIS3 expression in gastric cancer correlates with altered immune cell infiltration and poor prognosis. Its association with immune checkpoints implies a role in enabling tumors to evade the immune system. GLIS3 knockdown inhibits cancer growth and migration, and a GLIS3-based model accurately predicts survival risk, offering avenues for personalized treatment strategies. Those works uncovers novel biomarkers and genetic expressions, such as plasma tRF-33, HMGs, and GLIS3, that offer significant insights into the diagnosis, monitoring, and prediction of gastric cancer.

Understanding the genetic landscape of cancer is crucial in developing new avenues for diagnosis and treatment. Sun et al. investigated the expression profiles of long noncoding RNA (lncRNA) and mRNA in gastric cancer, identifying differences between early and advanced stages related to genes involved in tumorigenesis, including cell cycle and extracellular matrix organization. Key transcription factors E2F1, E2F4, and STAT2 were found to be associated with regulatory lncRNAs, and high expression of THBS2 may promote cancer progression. The study uncovers complex mechanisms and provides promising directions for future research and treatment.

The identification of prognostic biomarkers and key genes in various digestive system tumors is a critical step towards personalized medicine and targeted therapies. Song et al. found that SKA1-3, a complex of proteins involved in cell division, is a prognostic biomarker for human hepatocellular carcinoma. SKA1 and SKA3 were highly expressed in cancer patients, and this was associated with a worse prognosis. Ji et al., Zhu et al., and Jiang et al. have investigated key genes, such as ALKBH5, ITGB1, and KLHL14, that are associated with the prognosis of gastric cancer and lymphoma. Taken together, those studies provide insights into novel molecular markers and genetic pathways, which could enable novel approaches to prognosis, diagnosis, and treatment of cancers.

## 3 Machine learning and advanced modeling in cancer diagnose and research

Machine learning and deep learning have emerged as promising approaches to investigate tumors, with the potential to improve our understanding of cancer (15, 16). Zhou and Wang created a diagnostic model for digestive system tumors using gene set variation analysis and machine learning, offering a new approach to cancer screening and individualized treatment. They identified a prognostic model linked to the immune microenvironment and an intuitive assessment tool. Zeng et al. used deep learning algorithms to predict survival rates of gastric adenocarcinoma patients from a large dataset, outperforming traditional models with a good discriminatory ability. The DeepSurv model accurately predicted survival rates at various intervals, demonstrating the potential of deep learning for precise predictions in personalized treatment and clinical decision-making.

Early diagnosis is essential for fighting cancer, and innovative methods are key to progress. Machine learning and advanced modeling are being used to predict and diagnose cancer, with a special focus on discovering and evaluating biomarkers for early detection (17, 18). Zhang et al. found that the platelet-lymphocyte ratio, neutrophil-lymphocyte ratio, and systemic immune-inflammation index were significantly higher in gastric cancer patients, especially in early stages. The systemic immune-inflammation index had the highest diagnostic performance and combining it with the other ratios further improved efficiency. These indicators correlated with clinical progression like distant metastasis, emphasizing their potential in early diagnosis, prognosis evaluation, and treatment planning for gastric cancer. Chen et al. developed a predictive model using clinical data and gastric CT scans to identify patients with deficient mismatch repair in gastric cancer before surgery. Patients with this condition had larger tumors and a lower normalized tumor enhancement ratio in their scans. The study found that factors, like gender, age, tumor size, and this ratio were independent predictors. The model’s strong abilities in discrimination and calibration allow non-invasive identification, aiding treatment decisions for personalized care. Those works showcases the use of biological markers and modeling to improve early gastric cancer diagnosis.

Shu et al. analyzed single-cell sequencing data in esophageal squamous cell carcinoma, and found that SAA1+ malignant epithelial cells are key in metastasis and constructing a prognostic model to predict patient outcomes, thereby informing treatment. This work offers a new theoretical foundation for enhancing cancer treatment and improving patient outcomes. In a study conducted by Qiao et al., an analysis of 516 patients with advanced gastric cancer revealed correlations between the activity of the coagulation system and lymph node metastasis. They identified that platelet count, fibrinogen level, and maximum amplitude (a parameter of thromboelastographic assessing clot strength) were notably higher in those with positive lymph node metastasis. The maximum amplitude was an independent predictor of both metastasis and tumor stage. Those studies shedding light on the critical role of specific cellular characteristics and the coagulation system in esophageal and gastric cancer.

## 4 Immunotherapy and combination approaches, and an assessment of their effectiveness

Traditional therapy, immunotherapy, and combination therapy have all undergone substantial advancements, and research in these areas continues to be a prominent focus in the field.

Wang et al. conducted a study to investigate the impact of combination immunotherapy on gastrointestinal cancer patients of different age groups. Surprisingly, their findings revealed that younger patients experienced worse outcomes, whereas older patients demonstrated better responses to the treatment. Age did not impact immune-related side effects, indicating safety for older individuals, and these side effects correlated with better treatment success. Zhu et al. described a case of a 63-year-old woman with advanced gastric cancer who achieved complete remission with a combination of Tislelizumab and chemotherapy, highlighting the effectiveness of this approach and the importance of molecular markers in surgical decisions. Jiang et al. reported a case of a 59-year-old man with advanced gastric cancer who responded completely to a therapy combination including Camrelizumab, Apatinib, S-1, and Paclitaxel. The high expression of PD-L1, deficient mismatch repair, and correlations with gut microbiota were notable. Those studies collectively underline the potential of immunotherapy for gastrointestinal cancer, emphasizing factors like age, combined treatments, and molecular and microbial markers.

The identification and development of biomarkers and predictive models are becoming pivotal in the personalized treatment of gastrointestinal cancer. Zhang et al. conducted a review to investigate the predictive value of the neutrophil-to-lymphocyte ratio in survival prognosis for gastric cancer patients treated with immune checkpoint inhibitors. Analyzing data from nine studies and 806 patients, they found that a high ratio was linked with unfavorable overall survival, while a lower ratio was connected to an improved response rate. This research indicated that the neutrophil-to-lymphocyte ratio might be a promising biomarker for forecasting the prognosis and treatment response in gastric cancer patients receiving immune checkpoint inhibitors therapy. Wang et al. developed a prognostic model for locally advanced elderly esophageal cancer patients with positive EGFR who were treated non-surgically. They identified specific treatment, clinical stage, and performance score as key survival factors. The study showed the model’s effectiveness in predicting survival rates and revealed potential therapeutic benefits. Overall, those studies highlight the importance of biomarkers and predictive models in gastrointestinal cancer, offering insights into personalized treatment strategies.

Combination therapies are being actively researched and evaluated for advanced gastrointestinal cancers, with the goal of improving treatment outcomes and tailoring therapeutic strategies to the individual needs of each patient. Qu et al. assessed the efficacy of a combination therapy for advanced gastric adenocarcinoma or gastroesophageal junction adenocarcinoma in 28 patients. They found a response rate of 28.6% and identified male gender, liver metastases, and peritoneal metastases as risk factors. The combination therapy was generally well tolerated, making it as a potential treatment option for these patients. In a related investigation, Wang et al. investigated the combination of neoadjuvant immunotherapy with chemotherapy for locally advanced esophageal squamous cell carcinoma. They found the combined therapy significantly outperformed chemotherapy alone and identified specific immune cells as potential predictive markers for treatment success. Li et al. focused on 121 patients with advanced esophageal squamous cell carcinoma, examining the effects of radioimmunotherapy across various irradiation sites, including the brain. They found that brain irradiation led to stronger immune activation and identified specific blood indicators with predictive value for short-term treatment efficacy. Those studies illuminate the potentials and challenges of combination therapies, highlighting the complex relationships between treatment modalities and patient responses in gastric and esophageal cancers.

The real-world clinical practice and studies provide essential insights that bridge the gap between controlled research settings and everyday healthcare scenarios. In a clinical study conducted in real-world setting, Ohsawa et al. compared the effectiveness and safety of Nivolumab with Paclitaxel in treating patients with recurrent or advanced esophageal squamous cell carcinoma. Nivolumab showed a longer median survival time and fewer severe side effects than paclitaxel, particularly in second-line treatment, endorsing its use in such patients. Kowalchuk et al. studied the impact of post-surgical cardiopulmonary total toxicity burden on patients with esophageal cancer after trimodality therapy. They found that this measure could predict survival and complications, and identified age, chemotherapy toxicity, and radiotherapy techniques as influencing factors. The research suggests that optimizing preoperative care and reducing toxicity can improve outcomes. Collection, those reports in the field of gastrointestinal diseases have offered insights into the pathogenesis, diagnosis, and treatment of these disorders, emphasizing the need for personalized and comprehensive approaches, and contributing to advances in the field.

Understanding the underlying pathogenesis of cancer and identifying precise treatment targets remain central to the advancement of oncology, particularly in the complex landscape of gastrointestinal malignancies. Cachexia, a condition closely associated with cancer-related mortality, leads to progressive organ damage and dysfunction (19). Sui et al. identified increased expression of DUSP1 in the skeletal muscle of patients suffering from cancer cachexia and found that it inhibits muscle cell differentiation. This presents a possible target for treating this condition. Using patient information and statistical techniques, it may be possible to create predictive models to aid medical professionals in disease management. In a systematic review and meta-analysis, Noori et al. explored PD-L1 expression as a predictor of treatment success with ICIs in esophageal cancer. They found that patients with positive PD-L1 expression had a notable increase in survival time when treated with certain agents, providing guidance for patient-specific treatment. These studies highlight the potential of targeted approaches in understanding and treating complex conditions like cancer cachexia and esophageal cancer, pointing towards personalized therapies.

## 5 A glimpse into the future of therapeutic strategies

In the rapidly evolving field of gastrointestinal cancer treatment, researchers are investigating innovative approaches and novel strategies that could significantly improve therapeutic outcomes. Shi et al. studied the effect of different light strength on photodynamic therapy for gastrointestinal cancer. They found that intense light exposure led to quicker cell death, while weaker light was less effective, leading to possible tumor recurrence. This research provides an important reference for optimizing photodynamic therapy strategies for gastrointestinal tumors. Currently, radical surgical resection remains the primary treatment for esophageal cancer. However, esophageal anastomotic leak poses a serious postoperative complication after esophagectomy. Hu et al. investigated a novel approach for esophageal anastomotic leak treatment, involving the combined transplantation of mesenchymal stem cells and fibrin scaffold, which effectively promoted esophageal anastomotic leak closure and healing. Their porcine model experiment demonstrated the potential therapeutic efficacy of this method in treatment, offering promising prospects for improving patient outcomes and reducing treatment risks. Xiang et al. suggested Agrimol B as a potential drug for colorectal cancer, and Tan et al. reported a new technique for acute intestinal obstruction. Liu et al. found that the size of platelets could be a significant predictor for outcomes in esophageal squamous cell carcinoma. Larger platelets were linked to higher survival rates, and the study offers insights into whether postoperative chemotherapy is beneficial for certain patients.

As genomics technology continues to advance, the analysis of gene expression profiles coupled with the integration of clinical data, medical imaging, and laboratory information opens up new possibilities. The incorporation of these datasets with cutting-edge bioinformatics techniques, including machine learning algorithms, holds the promise of creating highly accurate and robust predictive models, which provide valuable support for medical decision-making. Research efforts will concentrate on the development of personalized therapeutic drugs and strategies to enhance treatment effectiveness and reduce unnecessary treatment risks. In-depth exploration of the mechanisms behind immunotherapies and chemotherapies will facilitate the discovery of new targets and combination treatment approaches. Overall, the medical field will continue its trajectory towards personalized medicine and intelligent healthcare, ultimately delivering more precise and effective medical services to patients.

## Author contributions

JH: Writing – original draft, Writing – review & editing. KZ: Writing – original draft, Writing – review & editing. QZ: Writing – review & editing. GP: Writing – review & editing. XH: Writing – review & editing. DC: Writing – review & editing.
